# Synergistic Effect of MC-LR and C-Terminal Truncated HBx on HepG2 Cells and Their Effects on PP2A Mediated Downstream Target of MAPK Signaling Pathway

**DOI:** 10.3389/fgene.2020.537785

**Published:** 2020-10-15

**Authors:** Chanchan Xiao, Fanbiao Mei, Guanhua Ren, Long Long, Maojian Chen, Xiang Fang, Jilin Li, Kezhi Li, Yanping Tang, Tianren Huang, Wei Deng

**Affiliations:** Guangxi Medical University Cancer Hospital, Nanning, China

**Keywords:** MC-LR, ctHBx, HepG2 cells, protein phosphatase PP2A, MAPK signaling pathway

## Abstract

C-terminally truncated hepatitis B virus (HBV) X (ctHBx) infection and exposure to microcystins-LR (MC-LR) can lead to human hepatitis and liver cancer, but the mechanism associated with their synergistically effects not been fully elucidated. The ctHBx (HBxΔ4 and HBxΔ32) lentivirus were constructed and transfected into the HepG2 cells. Then we investigated the function of MC-LR and ctHBx using the molecular biology approaches, including enzyme-linked immunosorbent assay, clone formation assay, scratch wound testing, transwell assays, carried out flow cytometry respectively to examine cell cycle and apoptosis in each group, and detected the related proteins of HBx, MEK/ERK/JNK/p38 in mitogen-activated protein kinase (MAPK) pathway and the downstream proteins such as cdc2, cdc25C, and p53 by western blotting. We found that the protein phosphorylase 2A (PP2A) enzyme activity in MC-LR and HBxΔ32/HBxΔ4 groups decreased more than in MC-LR and HBx group at the same time point and MC-LR concentration (*P* < 0.05). Meanwhile the proliferation, migration, invasion and colony formation capability of HepG2 cells were significantly enhanced in MC-LR and ctHBx groups (*P* < 0.05). In addition the proportion of S stage cells in the MC-LR-treated HBxΔ32/HBxΔ4 groups was significantly greater than that in the untreated groups (*P* < 0.05). Furthermore, the protein expression of MAPK signaling pathway including phospho-MEK1/2, ERKl/2, p38, and JNK were up-regulated by MC-LR and HBxΔ32, and the expression of cyclin-related proteins, including p53, cdc25C, and cdc2 were also activated (*P* < 0.05). Taken together, our findings revealed the essential significance of the MC-LR and ctHBx on the PP2A/MAPK/p53, cdc25C and cdc2 axis in the formation and development of HCC and identified MC-LR and ctHBx as potential causal cofactors of hepatocarcinogenesis.

## Introduction

Hepatitis B virus (HBV) infection, aflatoxin intake and microcystin-polluted drinking water are three major risk factors for hepatocellular carcinoma (HCC) (Yu, 1995; Mittal and El-Serag, 2013). A hepatitis B seroepidemiological survey conducted in 2006 showed that the prevalence rates of HBsAg in individuals aged 1 to 59 years and in children aged 1 to 4 years were 7.18 and 0.96%, respectively, and that approximately 93 million individuals in China are HBV carriers (Liang et al., 2013). According to the latest data for 2016, the estimated carrier rate of HBsAg in China was 6.1%, and approximately 86 million people had chronic HBV infection (Razavi-Shearer et al., 2018). Data from high incidence areas of liver cancer showed that the HBsAg carrying rates reached 20.21% in males aged 35–64 years and 13.18% in females aged 40–64 years (Zhang, 2006). It was reported that HBV infection leads to a wide spectrum of liver disease ranging from acute to chronic hepatitis, cirrhosis, and hepatocellular carcinoma due to the specific features of HBV virus (Liang, 2009).

The HBV genome is 3200 bp in length and includes four open reading frames (ORF), namely, C, S, P, and X (Motavaf et al., 2013), and as shown in the previous study, integration into the host genome occurs most frequently with the X gene, followed by the S, C, and P genes (Tarocchi et al., 2014). The HBx gene is 465 bp in length but is often partially deleted during integration such that this gene is often integrated into human chromosomes in a C-terminal truncated form (Wang et al., 2004). Hoare et al. (2001) observed that 10 (58.8%) of 18 patients with liver cancer have HBx deletion mutations. Various truncations in HBx have been verified by several studies, including the truncation of 20 or 35 amino acids of the c-terminally truncated HBV X (ctHBx) in liver cancer tissues (Chen et al., 2000; Ma et al., 2008). The results of our previous study revealed that the C-terminal deletions of four amino acids (HBxΔ4) and 32 amino acids (HBxΔ32) were the most common integration fragments of HBx, and these two ctHBx proteins have been shown to play key roles in HCC development (Zheng et al., 2015; Fang et al., 2017; He et al., 2017).

In terms of drinking water in HCC high-incidence areas of South-West China, the transformation of drinking water sources for humans and livestock has allowed local residents to no longer have to drink pond water. However, new problems have arisen with new water source, one of which is the generation of water blooms and the resulting production of microcystins (MCs) (Massey et al., 2018). To date, 90 MCs have been discovered (Pham and Utsumi, 2018), among which microcystin-LR (MC-LR) is the most common. In eutrophic waters, algal blooms occur and release microcystins, which become enriched in aquatic animals and enter the human body through dietary intake, harming human health (Pham and Utsumi, 2018; Xiao et al., 2018). A study of microcystins in Guangxi in 1996 demonstrated that the concentration of MCs in ditch pond water (30 water samples) and river water (eight water samples) was 160.5 and 231.3 pg/mL, respectively (Ueno et al., 1996). A recent survey revealed that the concentrations of MCs in source water and treated water supplies in high-incidence areas of Guangxi were 15.64 ± 2.08 and 14.42 ± 2.28 ng/L, respectively (Li et al., 2016). The above results indicate a high incidence of MC contamination in areas of Guangxi, but they do not take into account the ability of MCs and ctHBx to synergistically cause liver cancer in local residents.

The toxic effects of MC-LR shows obvious organ selectivity, with the liver being the primary target organ (Su et al., 2019). According to research reports, long-term exposure to MC-LR can induce human hepatitis and HCC (Ma et al., 2018a). MC-LR is a hepatotoxin that inhibits intracellular serine/threonine protein phosphatase 2A (PP2A), which regulates HepG2 cell differentiation, proliferation, invasion and the cell cycle (Falconer and Humpage, 2005; Jing et al., 2011; Sun et al., 2014; Yang et al., 2018). However, the mechanism underlying the involvement of MC-LR in hepatocarcinogenesis remains largely unknown. Studies have shown that MC-LR can activate the extracellular signal-regulated kinase (ERK), JNK, and P38 MAPK signaling pathways in human liver HL7702, Hek293 and Hela cells (Sun et al., 2011, 2014). Intriguingly, HBx reportedly activates MAPK signaling to promote oncogenesis in the early stages of chronic hepatitis B (Panteva et al., 2003). Therefore, changes in the MAPK signal transduction pathway may be relevant to the development of liver cancer. HBx mutants, particularly ctHBx, play a multifunctional carcinogenic role in the development of HBV-associated liver cancer, such as promoting cell proliferation and migration, and regulating the cell cycle and apoptosis (Iavarone et al., 2003; Motavaf et al., 2013). Therefore, there may be synergistic or antagonistic effect between MC-LR and ctHBx. Their interactions might influence the common MAPK signaling pathways involved and lead to more serious cytotoxicity or carcinogenesis. These issues need to be further explored. However, the involvement of MC-LR and ctHBx in carcinogenesis and progression is not well-understood, particularly with respect to regulation of PP2A enzyme activity to activate downstream MAPK signaling in hepatocellular carcinogenesis (Gong et al., 2015; Cheng et al., 2018), which is associated with changes in cell proliferation and invasion and in the cell cycle. This study is based on the hypothesis that MC-LR and ctHBx activate MAPK signaling to cause cancer by altering PP2A enzyme activity.

In this study, we investigated the effects of MC-LR and ctHBx on HepG2 proliferation, invasion, migration, and apoptosis and the cell cycle. Specifically, the MC-LR and ctHBx-induced changes in the cell cycle-related target proteins cdc2, cdc25C and p53, which are downstream of the MAPK pathway of PP2A, were explored. The results of this study indicated the importance of the MC-LR and ctHBx PP2A/MAPK/cdc25C and p53 axis in the formation and development of HCC and identified MC-LR and ctHBx as potential causal factors for hepatocarcinogenesis.

## Materials and Methods

### Cell Culture and MC-LR Exposure

The human hepatoma cell line HepG2 and SMMC-7721 were purchased from Zhongqiao Xinzhou Biotechnology Co., Ltd. and Biowing Biotechnology, Co., Ltd., respectively (Shanghai, China). These cells were cultured in Dulbecco’s modified Eagle’s medium (DMEM; Gibco, United States) supplemented with 10% fetal bovine serum (FBS; Gibco, United States), 100 μg/mL streptomycin (Hyclone, United States), and 100 U/mL penicillin (Hyclone, United States) in a humidified incubator with 5% CO_2_ at 37°C. Details of the cell genetic quality identification test report were shown in Supplementary Files 1, 2. MC-LR (purity > 95%) was purchased from the Beijing Solarbio Science & Technology, Co., Ltd., Beijing, it was dissolved in complete DMEM medium to a storage concentration of 100 μM, stock at −20°C until use.

### Stable Cell Line Construction

Construction of the HBx eukaryotic expression vector and the packaging of the virus were performed and confirmed by Sangon Biotech, Co., Ltd. (Shanghai, China). The packaged lentiviruses were named LV5-HBxΔ32, LV5-HBxΔ4, LV5-HBx, and LV-negative control (NC). HepG2 and SMMC-7721 cells transfection map, as shown in Supplementary File 3. Considering that HepG2 cell line originated from human hepatocarcinoma, it has many liver specific related functions, and can be passed on indefinitely under standard culture conditions like immortalized cells. It has been reported that the whole cell gene expression sequencing analysis and miRNA and mRNA expression profiling of HepG2 cells have been carried out, further confirming the similarity of some functions between HepG2 cells and human normal hepatocytes, which can be used for the experimental study of liver *in vitro* (Costantini et al., 2013; Bai et al., 2014). Therefore, HepG2 human hepatoma cell line was used in this study.

### Quantitative Reverse Transcription PCR

We utilized the online tools to design primers^1,2^, and the synthesis was completed by Guangzhou Aiji Biotechnology, Co., Ltd. Total RNA was extracted with Trizol reagent (Invitrogen, Carlsbad, CA, United States) according to the manufacturer’s instructions. Then cDNA was generated using a Reverse Transcriptase kit (Takara, Kusatsu, Japan). Then the cDNA was used as template to determine the level of mRNA expression. The relative expression of HBx was calculated and normalized to GAPDH using the 2^–ΔΔ*Ct*^ method with the following primers: HBXΔ4 (453 bp) F: 5′-GGTCTTTGTACTGGGAGGCT-3′, R: 5′-GGATCCATC CCTAGGTAGAT-3′; HBXΔ32 (369 bp) F: 5′-GCCCAAGGT CTTACATAAGA-3′, R: 5′-GGATCCATCCCTAGGTAGAT-3′; HBX (465 bp) F: 5′-GGAGGAGATTAGGTTAAAGGT-3′, R: 5′-GGATCCATCCCTAGGTAGAT-3′; and GADPH (1125 bp) F: 5′-AGAAGGCTGGGGCTCATTTG-3′, R: 5′-AGGGGCCATC CACAGTCTTC-3′.

### Western Blotting

After MC-LR (0–10 μM) exposure for (0–24 h), total proteins from HepG2 cells were extracted using RIPA buffer containing 0.1% proteinase inhibitor (Solarbio, Beijing, China; Lot No. P1260). We use preformed biofuraw precast gel (Tianneng, Guangzhou, China; Lot No. 180-8001H) in western blotting, which is applicable for proteins with molecular weights from 10 to180 kDa, equivalent to the gel concentrations range from 4 to 20%. The immunoreactive bands were visualized using an ECL WB Detection Reagent (Solarbio, Beijing, China) and were then scanned using a Bio-Rad Universal Hood III (Bio-Rad, Hercules, CA, United States). The results were analyzed with the imageJ software. The relative expression of the target protein content was valuated with the gray value ratio of target and GAPDH. Antibodies against HBx (Abcam, Cambridge, United Kingdom; Lot No. ab2741), MC-LR (Alexis, Inc., The Bronx, NY, United States; Lot No. ALX804320), GAPDH [Cell Signaling Technology, Inc. (CST), Boston, MA, United States; Lot No. 2118], p-ERK1/2 (CST; Lot No. 8544), ERK1/2 (CST; Lot No. 9252), p-JNK (CST; Lot No. 4668), JNK (CST; Lot No. 9252), p-p38 MAPK (CST; Lot No. 9211), p38 MAPK (CST; Lot No. 8690), p-cdc2 (CST; Lot No. 4539), cdc2 (CST; Lot No. 28439), p-cdc25C (CST; Lot No. 4901), cdc25C (CST; Lot No. 4688), p-p53 (CST; Lot No. 9289), and p53 (CST; Lot No. 2527) were used in this study.

We selected MC-LR and HBxΔ32 for verification of the downstream target of the MAPK signaling pathway of PP2A. To determine whether these proteins were affected by PP2A, cells were pretreated with the PP2A agonist d-erythro-sphingosine (DES; 10 μM) (Ambition Biotechnology, Beijing, China) for 12 h prior to exposing cells to MC-LR. DES was dissolved into 10 mM storage concentration with dimethyl sulfoxide (DMSO), stock at −20°C until use.

### Enzyme-Linked Immunosorbent Assay

After MC-LR exposure (0–10 μM) for 3–24 h, the PP2A level was determined using a PP2A ELISA Kit. According to the manufacturer’s instructions, the absorbance values at 450 nm of samples were read after incubating for 1 h.

### Scratch Wound Assay

Cells at 80% confluence were plated uniformly in complete medium in six-well plates. The scratch wounds (0 h) were imaged immediately after scratching, and after 24 h of incubation, the scratch wounds were visualized under a microscope (Leica DM4000B; Leica Microsystems, Wetzlar, Germany). All the experiments were performed in triplicate.

### Colony Formation Assay

Cells in the logarithmic phase of growth were seeded in a six-well plate at a density of 500 cells per well, and after MC-LR exposure (0 or 10 μM) for 24 h, they were cultured in an incubator at 37°C under an atmosphere with 5% CO_2_ for 14 days. The cells were carefully washed twice with PBS, fixed in 100% methanol for 15 min, and stained with 0.1% crystal violet for 15 min. The washing solution was slowly washed away with water and dried naturally in air. The number of clones larger than 50 cells were counted under a microscope, and the level of clone formation was then calculated.

### Transwell Assay

For the cell invasion and migration assays, transwell chambers (8-μm pores, Corning Incorporated, Corning, NY, United States) were coated with or without 50 μL of Matrigel (FBS-free medium diluted 1:6, BD Bioscience, Bedford, NY, United States) and then dried at 37°C for 4 h. The cells were suspended in FBS-free medium at a density of 2 × 10^5^ cells/mL. Subsequently, 100 μL of the cell suspension was seeded into the upper chamber, and 600 μL of medium containing 10% FBS was added to the bottom chamber. After 24 h of culture, the chamber was removed, and the cells in the upper chamber and Matrigel were wiped with a cotton swab, fixed in methanol for 20 min, stained with 0.1% crystal violet for 10 min, and washed twice with PBS. Photographs were obtained under an optical microscope. Subsequently, according to the manufacturer’s instructions, statistical analyses were conducted.

### Flow Cytometry

After MC-LR exposure (0–10 μM) for 24 h, the cell cycle and apoptosis of the cells were assessed using a cell cycle detection kit (Multiscience, Hangzhou, China) and an APC Annexin V and 7-AAD apoptosis detection kit (BD Bioscience, Bedford, NY, United States) according to the manufacturers’ recommended protocols.

### Statistical Analysis

The data were statistically analyzed by one-way ANOVA and *t*-tests using SPSS 22.0 software. The cell-based experiments were repeated at least three times, and the results are presented as the means ± standard deviations (SDs). Differences with *P* < 0.05 were considered significant.

## Results

### Intracellular Expression of HBxΔ32, HBxΔ4, and HBx

**FIGURE 1:**
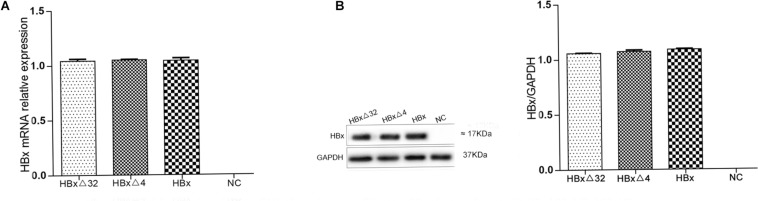
Expression of HBx mRNA **(A)** and protein **(B)** in HepG2 cells transfected with HBxΔ32, HBxΔ32, HBx and empty vector. HBxΔ32, HBxΔ4, HBx and empty vector [negative control (NC)] were transfected to HepG2 cells respectively. The mRNA and protein expression levels of HBx and the two mutants were assessed by qRT-PCR **(A)** and Western blot **(B)**. Except for NC group, the HBxΔ32, HBxΔ4, and HBx groups were demonstrated that the transfected genes and proteins were efficiently expressed and shown no significant difference. As the picture **(B)** showed, the molecular weights of HBxΔ32, HBxΔ4, and HBx are equal to or very closed to 17 kDa. The gel used for Western Blot are precast and applicable for proteins with molecular weights from 10 to 180 kDa, equivalent to the gel concentrations range from 4 to 20%.

The expression of HBxΔ32, HBxΔ4, and HBx in HepG2 cells was assessed by RT-qPCR and western blotting. The HBxΔ32, HBxΔ4, and HBx genes were successfully amplified from cDNAs isolated from the three transfected cell populations (Figure 1A), whereas no HBX amplification was observed using cDNA from control cells (Figure 1A). The western blot results showed the HBx-specific band (17 kDa) in the protein samples from the three transfected cell populations, whereas no HBx band was observed with the protein samples from the control cells (Figure 1B). These results confirmed that intracellular HBxΔ32, HBxΔ4, and HBx were successfully expressed and that the transfected HepG2 cell lines were available for subsequent cell function experiments.

### MC-LR and ctHBx Effect on PP2AA Activity of Intracellular Protein Phosphatase and Detection of MC-LR Entrancen Into the Cells

**FIGURE 2:**
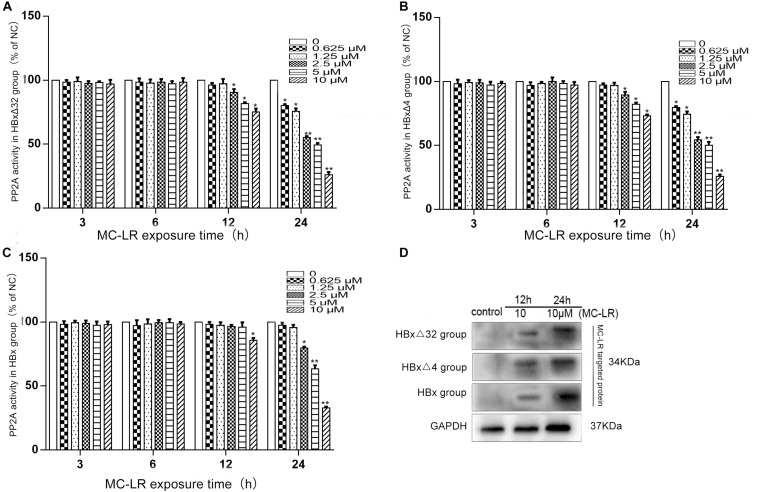
The alteration of PP2A activity in HepG2 cells after MC-LR exposure. The HBxΔ32, HBxΔ4, HBx, and NC transfected HepG2 cells were treated with MC-LR at various concentrations, and the PP2A enzyme activity were examinated by ELISA after 3, 6, 12, and 24 h. The results showed that the PP2A enzyme activity in HBxΔ32 **(A)** and HBxΔ4 **(B)** groups both began to decrease when MC-LR ≥ 2.5 μM at 12 h and ≥ 0.625 μM at 24 h; While in HBx group **(C)**, the PP2A enzyme activity began to decrease until MC-LR = 10 μM at 12 h and ≥ 2.5 μM at 24 h. **(D)** The WB assay showed that MC-LR entered HepG2 cells and targeted to putative proteins weighted 34 kDa in HBxΔ4, HBxΔ32, and HBx groups. Bar graphs indicate means ± standard deviation from three independent experiments. *P < 0.05, **P < 0.01 vs. 0 μM MC-LR (Complete DMEM medium, the same as below).

The effects of MC-LR and ctHBx on protein phosphatase PP2A activity in HepG2 cells are shown in Figures 2A–C. As shown in Figure 2A, compared with that observed in the control group, the PP2A activity in HBxΔ32 cells decreased to 90, 81, and 75% after being treated with 2.5, 5, and 10 μM MC-LR for 12 h, respectively. After 10 μM MC-LR treatment for 24 h, as the time of action of the toxin increased, the activity of PP2A decreased significantly, with the lowest observed activity being approximately 26% that of the control. As shown in Figure 2B, compared with the control group, the PP2A activity in HBxΔ4 cells decreased to 89, 82, and 73% after being treated with 2.5, 5, and 10 μM MC-LR for 12 h, respectively. After 10 μM MC-LR treatment for 24 h, as the time of action of the toxin increased, the activity of PP2A decreased significantly, with the lowest observed activity being approximately 25% that of the control. As shown in Figure 2C, compared with the control group, the PP2A activity in HBx cells decreased to 85% after being treated with 10 μM MC-LR for 12 h. After 10 μM MC-LR treatment for 24 h, as the time of action of the toxin increased, the activity of PP2A decreased significantly, with the lowest observed activity being approximately 32% of the control. The inhibition of PP2A enzyme activity by MC-LR and HBxΔ32 or HBxΔ4 was not significantly different at the same time point and concentration (*P* > 0.05), and the inhibition of MC-LR and HBxΔ32 or HBxΔ4 on PP2A enzyme activity at the same time point and concentration was greater than that observed for MC-LR and HBx, and the difference was significant (*P* < 0.05). As shown in Figure 2D, the western blot results showed that MC-LR entered HBxΔ4, HBxΔ32, and HBx cells in a time-dependent manner. In addition, a specific band represented the MC-LR integrated proteins at 34 kDa was observed.

### MC-LR and ctHBx Enhance the Migration, Invasion, and Colony Formation Capabilities of HepG2 Cells

**FIGURE 3:**
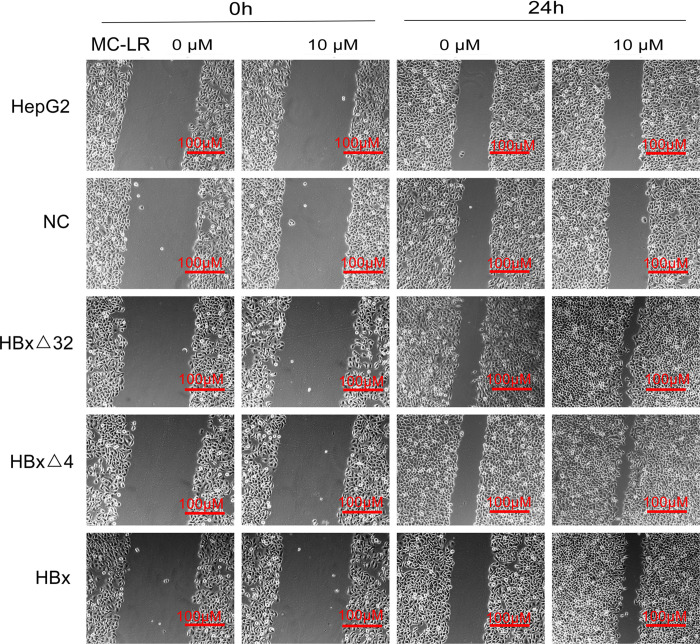
Effect of MC-LR and ctHBx on the motility ability of HepG2 cells. Scratch wound analysis of cells at 0 and 24 h after treating with 10 μM MC-LR. The fold changes in the scratch of the HBxΔ32, HBxΔ4, and HBx groups exposed to MC-LR (10 μM) were significantly higher than those of the NC and HepG2 groups (*F* = 28.351, *P* = 0.001), the motility ability of HBxΔ32 and HBxΔ4 groups were stronger than HBx group (HBxΔ32 vs. HBx *P* = 0.001; HBxΔ4 vs. HBx *P* = 0.001).

**FIGURE 4:**
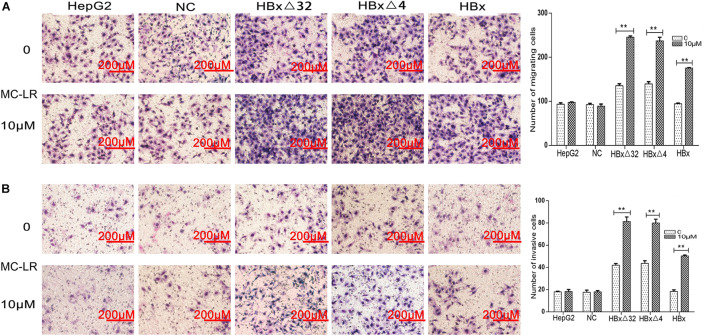
Effect of MC-LR and ctHBx on the migration and invasion abilities of HepG2 cells. A transwell assay was performed to investigate the effect of MC-LR and ctHBx on the migration and invasion of HepG2 cells. **(A)** The results showed that the migration between HBxΔ32, HBxΔ4, and HBx groups were statistically difference (*F* = 52.074, *P* = 0.001), HBxΔ32 and HBxΔ4 groups were stronger than HBx group (HBxΔ32 vs. HBx *P* < 0.001; HBxΔ4 vs. HBx *P* < 0.001). **(B)** When comparing the invasion, the statistical differences were also found (*F* = 31.177, *P* = 0.001), HBxΔ32 and HBxΔ4 groups were stronger than HBx group (HBxΔ32 vs. HBx *P* < 0.0001; HBxΔ4 vs. HBx *P* = 0.001). Bar graphs indicate means ± standard deviation from three independent experiments. **P < 0.01 vs. 0 μM MC-LR.

**FIGURE 5:**
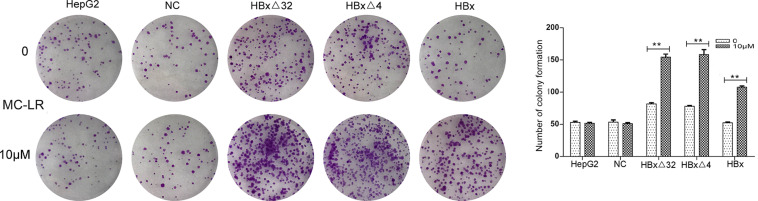
Effect of MC-LR and ctHBx on the colony formation capabilities of HepG2 cells. The colony forming ability between HBxΔ32, HBxΔ4, and HBx cells incubated with 10 μM MC-LR were compared, it was found that the colony forming ability of these three groups were statistically difference (*F* = 26.948, *P* = 0.001), HBxΔ32 and HBxΔ4 groups were stronger than HBx group (HBxΔ32 vs. HBx *P* = 0.001; HBxΔ4 vs. HBx *P* = 0.001). Bar graphs indicate means ± standard deviation from three independent experiments. **P < 0.01 vs. 0 μM MC-LR.

The effects of MC-LR and ctHBx on the motility of HepG2 cells were evaluated using scratch wound assays. The results showed that the MC-LR-treated HBxΔ32, HBxΔ4, and HBx groups tended to exhibit increased migration compared with that observed in the MC-LR-treated NC and HepG2 groups (Figure 3). Transwell assay were then performed to investigate the effect of MC-LR and ctHBx on the migration (Figure 4A) and invasion (Figure 4B) of HepG2 cells. After treatment for 24 h, the migration and invasion abilities of the MC-LR (10 μM)-treated HBxΔ32, HBxΔ4, and HBx groups were significantly increased compared with that in the MC-LR-treated NC and HepG2 groups. In addition, although the number of migrated and invaded cells in the MC-LR-treated HBxΔ32 and HBxΔ4 groups were not significantly different, the number of invaded and migrated cells in the MC-LR-treated HBxΔ32, HBxΔ4, and HBx groups were significantly higher than those the untreated groups. These results confirmed that MC-LR and ctHBx synergistically promote the migration and invasion of HepG2 cells *in vitro*. A clone formation assay was then performed to further verify the effects of MC-LR and ctHBx on cell proliferation (Figure 5). The clone numbers of the MC-LR-treated HBxΔ32, HBxΔ4, and HBx groups were significantly increased compared with those observed in the MC-LR-treated NC and HepG2 groups, and the clone numbers from the MC-LR-treated groups were significantly higher than those observed in the untreated groups. These results confirmed that MC-LR and ctHBx synergistically promote the proliferation of HepG2 cells *in vitro*.

### MC-LR and ctHBx Increase the Percentage of Cells in the S Phase of the Cell Cycle

**FIGURE 6:**
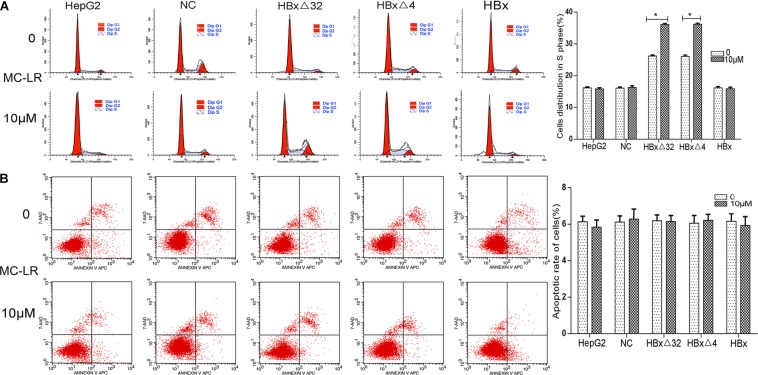
Effect of MC-LR and ctHBx on the cell cycle and apoptosis of HepG2 cells. The cell cycle analysis showed that the HBxΔ32 and HBxΔ4 groups exposed to MC-LR (10 μM) included a greater proportion of cells in the S phase than the NC, HBx, and HepG2 groups exposed to MC-LR (HBxΔ32 vs. HBx *P* < 0.05; HBxΔ4 vs. HBx *P* < 0.05). **(A)** The apoptosis rates of the HBxΔ32, HBxΔ4, and HBx groups exposed to MC-LR (10 μM) were not significantly different from those of the NC and HepG2 groups exposed to MC-LR (*F* = 0.119, *P* = 0.890). **(B)** Bar graphs indicate means ± standard deviation from three independent experiments. *P < 0.05 vs. 0 μM MC-LR.

The role of MC-LR and HBxΔ32, HBxΔ4, or HBx on regulation of the cell cycle and apoptosis in HepG2 cells was explored through flow cytometry analysis (Figure 6). The cell cycle analysis results showed that the MC-LR (10 μM)-treated HBxΔ32 and HBxΔ4 groups exhibited a greater proportion of cells in the S phase than that observed in the MC-LR-treated NC, HBx, and HepG2 groups (Figure 6A). In addition, the proportion of cells in the S phase in the MC-LR-treated HBxΔ32 and HBxΔ4 groups was not significantly different, whereas the proportion of cells in the S for the MC-LR-treated groups was significantly greater than that observed in the untreated HBxΔ32 and HBxΔ4 groups. These results confirmed that MC-LR and HBxΔ32 or HBxΔ4 synergistically promote the proliferation of HepG2 cells *in vitro*. The apoptosis rates of the MC-LR (10 μM)-treated HBxΔ32, HBxΔ4, and HBx groups were not significantly different from those observed in the MC-LR-treated the NC and HepG2 groups (Figure 6B).

### MC-LR and HBxΔ32 Activate the MAPK Signaling Pathway

**FIGURE 7:**
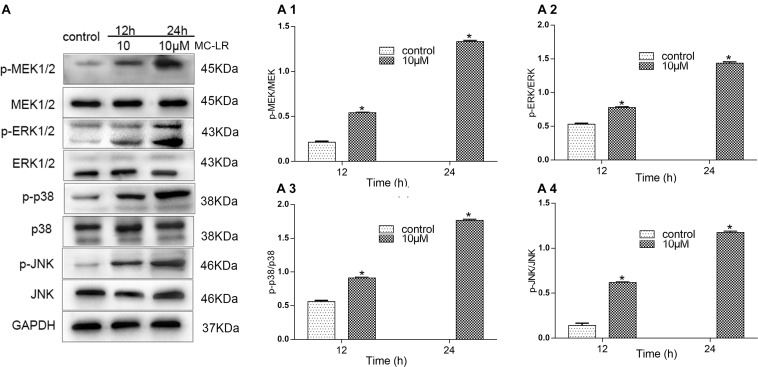
MC-LR and HBxΔ32 activate the MAPK signaling pathway. The HBxΔ32 cells were incubated with 10 μM MC-LR for 12 and 24 h. The protein expression of MAPK was detected by a WB and the representative blots are shown as picture **(A)**. Bar graphs indicate means ± standard deviation from three independent experiments. Values were obtained by comparing phosphorylated bands with total protein bands, and the results were shown as **(A1)** Phospho-MEK1/2 (p-MEK) and MEK; **(A2)** phospho-ERKl/2 (p-ERK) and ERK; **(A3)** phospho-p38 (p-p38) and p38; **(A4)** phospho-JNK (p-JNK) and JNK. *P < 0.05 vs. 0 μM MC-LR.

In this study, we observed that HBxΔ32 cells treated with MC-LR (0 and 10 μM) were most significantly inhibited for PP2A enzyme activity at two time points (12 and 24 h) (Figure 2A), which were subsequently used to assess the phosphorylation levels of the MAPK signaling pathway proteins MEK1/2, ERK1/2, p38, and JNK (Figure 7A). The MEK1/2, ERK1/2, p38, and JNK phosphorylation levels in HBxΔ32 group treated with MC-LR (10 μM) for 12 h were significantly increased compared with those observed in the cells with HBxΔ32 alone, which exhibited very low phosphorylation levels for these proteins. Furthermore, the phosphorylation levels of these proteins was dependent on the MC-LR exposure time. These results confirm that MC-LR and HBxΔ32 synergistically activate the MAPK signaling pathway.

### MC-LR and HBxΔ32 Modulates the Phosphorylation of p53, cdc25C, and cdc2 Proteins Through the Activity of PP2A

**FIGURE 8:**
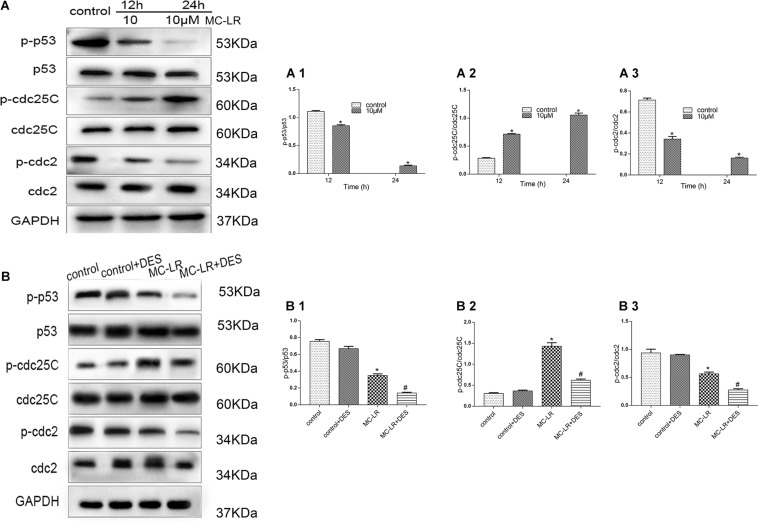
MC-LR and HBxΔ32 modulate the phosphorylation of the p53, cdc25C, and cdc2 proteins through the activity of PP2A. The HBxΔ32 transfected HepG2 cells were incubated with 10 μM MC-LR for 12 and 24 h, then the protein expression of p53, cdc25C, and cdc2 were detected by WB and the representative blots are shown as picture **(A)**. Bar graphs indicate means ± standard deviation from three independent experiments. Values were obtained by comparing phosphorylated bands with total protein bands, and the results were shown as **(A1)** Phospho-p53 (p- p53) and p53; **(A2)** phospho-cdc25C (p-cdc25C) and cdc25C; **(A3)** phospho-cdc2 (p-cdc2) and cdc2. The HBxΔ32 transfected HepG2 cells were pre-incubated with 10 μM DES for 12 h and then exposed to 10 μM MC-LR for 24 h, the control group was not exposed to MC-LR, then the protein expression of p53, cdc25C, and cdc2 was detected by WB and the representative blots are shown as picture **(B)**. Bar graphs indicate means ± standard deviation from three independent experiments. Values were obtained by comparing phosphorylated bands with total protein bands, and the results were shown as **(B1)** Phospho-p53 (p- p53) and p53; **(B2)** phospho-cdc25C (p-cdc25C) and cdc25C; **(B3)** phospho-cdc2 (p-cdc2) and cdc2. *P < 0.05 vs. 0 μM MC-LR; ^#^*P* < 0.05 vs. 0 μM MC-LR and DES.

To investigate the effects of MC-LR and HBxΔ32 on the cell cycle, we examined proteins involved in cell cycle regulation, including p53, cdc25C, and cdc2 (Figure 8). After MC-LR treatment of HBxΔ32 cells, the levels of phosphorylated p53 and cdc2 proteins were decreased compared with those observed in the control group, the MC-LR (10 μM)-treated HBxΔ32 cells began to exhibit decreased phosphorylation of these proteins at 12 h and was dependent on MC-LR exposure time, while the level of phosphorylated cdc25C protein increased in a time-dependent manner 12 h after MC-LR exposure (Figure 8A). HBxΔ32 cells were pretreated with the PP2A protein phosphatase agonist DES (10 μM) for 12 h and then exposed to 10 μM MC-LR for 24 h. The control group was not treated with MC-LR. After DES intervention, the levels of phosphorylated p53, cdc25C, and cdc2 in the MC-LR + DES group were lower than those observed in the MC-LR group (Figure 8B). These results indicate that the phosphorylation levels of p53 and cdc2 are regulated by the PP2A enzyme and that the PP2A protein phosphatase agonist DES can attenuate the phosphorylation-promoting effect of cdc25C protein induced by MC-LR.

## Discussion

It was reported that the intake of MC-LR is a risk factor leading to HCC (Ueno et al., 1996). Our previous studies explored the integration site and target genes of HBX in the chromosomes of host liver cancer cells, and the results revealed that ctHBx (HBxΔ32 and HBxΔ4) is a commonly integrated fragment in liver cancer tissues (Zheng et al., 2015; Fang et al., 2017; He et al., 2017). However, the mechanism of the synergistic activity of MC-LR and ctHBx in HCC remains poorly understood. In this study, we constructed an *in vitro* cell model consisting of common integration fragments of HBx and explored the effect of MC-LR and ctHBx on the biological activity of HCC cells and the possible mechanism of through which MC-LR and HBx integration leads to the occurrence and development of HCC at the cellular level.

High molecular weight MC-LR cannot easily to diffuse into cells through the plasma membrane with the help of any transporter in the cell membrane (Fischer et al., 2005). Therefore, it is indispensable for this study to identify whether MC-LR entered HepG2 cells. The western blot results showed that MC-LR entered HBxΔ4, HBxΔ32, and HBx cells in a time-dependent pattern (Figure 2D). MC-LR is a well-known hepatotoxin that can greatly inhibit intracellular PP2A activity, which regulates HepG2 cell proliferation, differentiation, and invasion as well as the cell cycle (Sun et al., 2014; Lyons et al., 2018). Similarly, ctHBx could also affect the proliferation migration and invasion capabilities of HepG2 cells (Iavarone et al., 2003; Zhang et al., 2008). In this study, MC-LR and ctHBx were shown to synergistically inhibit the activity of intracellular PP2A in a dose- and time-dependent manner. These results indicate that this PP2A inhibition can lead to excessive phosphorylation of a large number of proteins in cells, and the hyperphosphorylation of these proteins is likely to be associated with the mechanism by which MC-LR and ctHBx function in HCC (Humpage et al., 2000; Mezhoud et al., 2008).

The observed effects of MC-LR and ctHBx on cell clone numbers in the present study showed that the MC-LR-treated HBxΔ32, HBxΔ4, and HBx groups exhibited increased levels of cell proliferation compared with the MC-LR-treated NC and HepG2 groups. Ma et al. reported that the exposure of HepG2 cells to MC-LR (0.1 nM to 10 μM) for 48 h did not significantly affect cell proliferation (Ma et al., 2017, 2018a, b). Studies have shown that exposure to low concentrations of MC-LR (0.01–100 nM) did not affect the viability of HepG2 cells and may be related to the tolerance of HepG2 cells to low concentrations of MC-LR (Jasionek et al., 2010). This finding was consistent with those obtained in our studies, where in the MC-LR (10 μM)-treated NC and HepG2 cell groups, no cell proliferation, migration and invasion changes were observed. Regarding studies on the effect of ctHBx on cell proliferation and its mechanism, it was reported that HBxΔ35 and HBxΔ14 could inhibit the transcriptional activity of some miRNAs by binding to the promoters of genes with a growth inhibitory function and may thereby increase the proliferation of tumor cells (Yip et al., 2011). The results of the present study revealed that the number of clonal cells in the MC-LR-treated HBxΔ32, HBxΔ4, and HBx groups were significantly higher than that observed in the untreated groups. This finding demonstrated that MC-LR and ctHBx synergistically promote the proliferation of HepG2 cells and that the last four to 32 amino acids in the C-terminus of HBx may not be the primary functional domain affecting the proliferation of HCC cells. In contrast, MC-LR and ctHBx could also affect the migration and invasion capabilities of HepG2 cells. In this study, the migration and invasion abilities of the MC-LR (10 μM)-treated HBxΔ32, HBxΔ4, and HBx groups were significantly increased compared with that observed in MC-LR-treated NC and HepG2 groups. In addition, the number of invaded and migrated cells from the MC-LR-treated HBxΔ32, HBxΔ4, and HBx groups was significantly higher than that observed in the untreated groups. Studies have reported that HepG2 cells exposed to MC-LR (0.25–2.5 μM) for 24 h were not induced for cell migration and invasion (Boaru et al., 2006). Sze et al. (2013) observed that HBxΔ24 can increase the invasion and metastasis abilities of tumor cells by enhancing C-Jun transcriptional activity and increasing the transcription of matrix metalloproteinases. Zhang et al. (2008) observed that HBxΔ27 can activate the arachidonic acid-catalyzing enzyme 5-LOX osteopontin and eventually promotes the migration of liver cancer cells. Taken together, these findings support those of his study showing that MC-LR and ctHBx synergistically promote the migration and invasion of HepG2 cells *in vitro*.

In this study, the role of MC-LR and HBxΔ32, HBxΔ4, or HBx on the regulation of the cell cycle and apoptosis in HepG2 cells was explored through flow cytometry analysis. The results showed that the percentage of S-phase cells in the MC-LR-treated HBxΔ32 and HBxΔ4 groups was higher than that observed in the control group. An earlier study showed that treatment of HepG2 cells with MC-LR (0.1 nM to 10 μM) for 24 h did not affect HepG2 cell cycle and apoptosis (Ma et al., 2017, 2018a). These *in vitro* results showed that the assayed MC-LR treatment concentrations and times were inadequate to affect the cell cycle and apoptosis. Furthermore, it is also possible that MC-LR is metabolized by cells after entering HepG2 cells, and insufficient amounts cannot affect the biological function of cells. Several studies have shown that ctHBx alters cell cycle regulators, including decreasing the expression of p15 and p16, decreasing DNA synthesis, and increasing the expression of p21, p27, cyclin D1, and cyclin E (Qiao et al., 2001; Gearhart and Bouchard, 2010, 2011). Furthermore, the proportion of cells in the S phase in the MC-LR-treated HBxΔ32 and HBxΔ4 groups was significantly greater than that observed in the untreated groups. Taken together, these results suggest that MC-LR and HBxΔ32 or HBxΔ4 synergistically promote the cell cycle in hepatocytes. Furthermore, this effect, which is mediated by cell cycle regulation, may have long-term effects on hepatocyte physiology, alter hepatocyte proliferation-related pathways, and contribute to the development of HCC.

To construct a downstream target and pathway detection model to assess the effects of MC-LR on PP2A, we selected MC-LR and HBxΔ32 for verification of the downstream targets of the MAPK signaling pathway of PP2A. The MAPK signaling pathway is an important cell signal transduction pathway that regulates cell proliferation, differentiation, and apoptosis and the cell cycle (Obata and Noguchi, 2004). In this study, we examined the phosphorylation levels of the MAPK family members MEK1/2, ERK1/2, JNK, and p38 and confirmed that MC-LR and HBxΔ32 could significantly increase the phosphorylation levels of these proteins, whereas the phosphorylation levels of these proteins in the control groups were low and decreased in an exposure time-dependent manner MC-LR. Studies have shown that MC-LR treatment can activate three MAPK signaling pathways (ERK, JNK, and P38) in the human liver cell lines HL7702, Hek293, and Hela (Zhang et al., 2008; Gearhart and Bouchard, 2010; Sun et al., 2014). A previous study revealed that cytoplasmic HBx appears to modulate Ras/MAPK signaling (Panteva et al., 2003). HBx activates the Ras/Raf/ERK pathway, which leads to transcriptional transactivation and quiescent cell transformation (Kim et al., 2001). Thus, the results of this study confirmed that MC-LR and HBXΔ32 had a synergistic effect on the phosphorylation of these MAPK proteins, magnifying signaling of a common MAPK pathway.

Because our flow cytometry results confirmed the important roles of MC-LR and HBxΔ32 in the regulation of the cell cycle, we hypothesized that the changes in the phosphorylation levels of MEK1/2, ERK1/2, p38, and JNK may be associated with the cell cycle-associated proteins p53, cdc2, and cdc25C. Previous studies demonstrated that phosphorylated cdc25C is located in the cytoplasm and fails to activate the related cyclin cdc2, ultimately inhibiting cell cycle progression (Kumagai and Dunphy, 1999). In this study, we detected an increase in the levels of phosphorylated cdc25C compared to that observed in the control group, whereas decreased levels of p53 and cdc2 phosphorylation were observed compared to that detected in the control group. Based on these results, we conclude that phosphorylation of cdc2 is not only regulated by the cyclin cdc25C but may also be regulated by other signaling pathways. Li et al. (2006) observed that the dephosphorylation of p53 results in a decrease in p53 transcription, which in turn results in negative regulation of the p53-dependent death pathway and the promotion of cell proliferation. These results shown that MC-LR and HBxΔ32 affect the expression the cyclin-related proteins p53, cdc25C and cdc2, which play important regulatory roles in the mitosis of eukaryotic cells, the promotion of the cell cycle through the dephosphorylation and activation of cell cycle-dependent enzymes and in embryogenesis and tumorigenesis. Based on the above results, to confirm that the changes in p53, cdc25C and cdc2 phosphorylation levels were the result of changes in PP2A activity, we used the PP2A protein phosphatase agonist DES. After DES intervention, the levels of phosphorylated p53, cdc25C, and cdc2 in the MC-LR + DES group were lower than those observed in the MC-LR group. These results indicate that the phosphorylation levels of p53 and cdc2 are regulated by the PP2A enzyme and that the PP2A protein phosphatase agonist DES can attenuate the phosphorylation-promoting effect of cdc25C induced by MC-LR.

In summary, in this study, we demonstrated that MC-LR and ctHBx can synergistically promote the proliferation, migration and invasion of HepG2 cells via the PP2A/MAPK/p53, cdc25C, and cdc2 axis and play important roles in hepatocarcinogenesis. However, our study has several limitations. First, although the experiments using HepG2 cells have revealed the relationship between MC-LR, HBx, and MAPK/PP2A pathway, we need to verify the current results using other hepatoma cell lines and the immortalized hepatocytes co-expressing MC-LR and HBx. Second, No *in vivo* experiments using an animal model were performed, which is especially important to study the effects of low-level long-term exposure to systematically study the carcinogenic effects and mechanisms of MC-LR on hepatocytes and comprehensively evaluate the safety risks of MC-LR and its impact on the health of susceptible populations. Therefore, the specific carcinogenic mechanism associated with the MC-LR and ctHBx needs to be further studied.

## Data Availability Statement

The raw data supporting the conclusions of this manuscript will be made available by the authors, without undue reservation, to any qualified researcher.

## Author Contributions

WD and TH designed the study and provided the funds. CX, LL, FM, GR, MC, and XF performed the experiments. CX, WD, LL, XF, JL, KL, and YT analyzed the data. CX wrote the manuscript. All authors contributed to the article and approved the submitted version.

## Conflict of Interest

The authors declare that the research was conducted in the absence of any commercial or financial relationships that could be construed as a potential conflict of interest.

## Footnotes

1 https://www.ncbi.nlm.nih.gov/gene/

2 https://pga.mgh.harvard.edu/primerbank/
