# Supplementary material for: Synergistic Effect of MC-LR and C-Terminal Truncated HBx on HepG2 Cells and Their Effects on PP2A Mediated Downstream Target of MAPK Signaling Pathway
Source: Front Genet. 2020 Oct 15;11:537785. doi: 10.3389/fgene.2020.537785 (PMC7593820; doi:10.3389/fgene.2020.537785)
Supplement: Supplementary file 1 [file Data_Sheet_1.PDF]

Cell genetic quality identification test report

Sample name: Cell line

Test type: STR genotype test

Sample number：

Table 1 Sample number

Customer sample number	Company number
427	20180615-01

Sample quantity: 1

Sample traits: Cell line

Test item: STR

Submission unit: Shanghai Zhongqiao Xinzhou Biotechnology Co., Ltd.

Detection method: DNA was extracted with Axygen's genomic extraction kit, amplified using a 20-STR amplification protocol, and the STR locus and sex gene Amelogenin were detected on an ABI3730XL genetic analyzer.

Test results

（一）Check the basic situation：

Table 2 Sample genotype test results

	Multiple alleles	Matching cell lines	Cell bank	EV value	Match description
20180615-01	Yes	Hep-G2, Hep-G2/ 2.2.15, etc.	DSMZ	0.94	Basic match

Note:1、Multiple alleles refer to the phenomenon of three or more alleles.

2、The results of cell typing were good.

(二) Description of each sample

20180615-01:The DNA typing of this cell found a basic matching cell line in the search. The DSMZ database showed that the cell names were Hep-G2, Hep-G2/2.2.15, etc., and the cell number corresponded to 180. This test was performed in this cell line Multiple alleles were found.

(三) Sample typing results

Genotyping results of STR site and Amelogenin site of cell 20180615-01						
Loci	STR information of submitted cells			Cell bank cell STR information		
	Customer sample number:427			Cell lines:Hep-G2, Hep-G2/2.2.15		
	Allele1	Allele2	Allele3	Allele1	Allele2	Allele3
D5S818	11	12		11	12	
D13S317	13	13		9	13	
D7S820	10	10		10	10	
D16S539	12	13		12	13	
VWA	17	17		17	17	
TH01	9	9		9	9	
AMEL	X	Y		X	Y	
TPOX	8	9		8	9	
CSF1PO	10	11		10	11	
D12S391	21	25				
FGA	22	25	26			
D2S1338	19	20				
D21S11	29	31				
D18S51	13	14				

D8S1179	15	16				
D3S1358	15	16				
D6S1043	13	13				
PENTAE	15	20				
D19S433	15.2	15.2				
PENTAD	9	13				

Other notes:

(1) Typing scheme and site distribution

Attached table: experimental scheme and site

	Scheme 1	Scheme 2	Scheme 3	Scheme 4
1	TH01	TPOX	D3S1358	AMEL
2	D12S391	VWA	D13S317	D5S818
3	D7S820	D8S1179	D6S1043	D2S1338
4	CSF1PO	PENTAD	D16S539	D21S11
5	FGA		D19S433	D18S51
6	PENTAE			

Certificate of STR Analysis

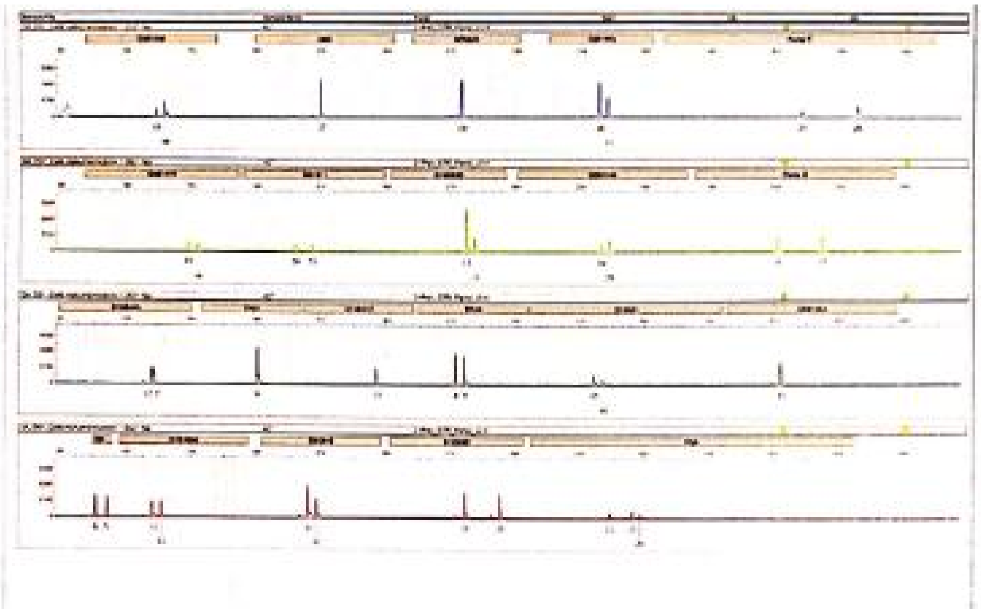

Date of issue

June 2018
