# Supplementary material for: Synergistic Effect of MC-LR and C-Terminal Truncated HBx on HepG2 Cells and Their Effects on PP2A Mediated Downstream Target of MAPK Signaling Pathway
Source: Front Genet. 2020 Oct 15;11:537785. doi: 10.3389/fgene.2020.537785 (PMC7593820; doi:10.3389/fgene.2020.537785)
Supplement: Supplementary file 2 [file Data_Sheet_2.PDF]

SHANGHAI BIOWING BIOTECHNOLOGY Co. LTD
Your Intimate Partner in Bioscience!

Cell Line Authentication Service

STR Profiling Report

Sample Type: Cell Line

Sample from: Guangxi Medical University Cancer Hospital

Testing Method: STR Genotyping

Report Time: May 28, 2018

COMPANY STATEMENT

1. THIS REPORT IS ONLY RESPONSIBLE FOR THE SAMPLES ANALYZED.
2. THE TESTING RESULTS AND THE ORGANIZATION NAME WILL NOT BE USED FOR ADVERTISEMENT, COMMERCIAL EXHIBITIONS, COMMERCIAL PERFORMANCE AND OTHER COMMERCIAL ACTIVITIES.
3. OBJECTIONS SHOULD BE RAISED WITHIN FIFTEEN DAYS AFTER THE RECEIPT OF THIS REPORT.
4. THE PAPER REPORT WITH CONTENT ALTERING, ADDING OR WITHOUT THE STAMPED SEAL OF THE COMPANY ARE INVALID.

Testing Company: Shanghai Biowing Applied Biotechnology Co. Ltd

Address: Room 4F, 8 th Buiding,Guiguo Garden,NO.471 Guiping Road,Caohejing Development Zone,Shanghai

Tel: +86-021-33559491

Contact: YiQun Chen

E-mail: biowing@vip.163.com

Cell Line Authentication – STR Profiling Report

Sample code

Table 1. Sample Code

Customer's code	Company Code
7721	20180519-04

Sample Number:1

Sample Type: Cell line

Testing Type: STR

Sample From: Guangxi Medical University Cancer Hospital

Testing Method:

DNA was extracted by a commercial kit from CORNING (AP-EMN-BL-GDNA-250G). The twenty STRs including Amelogenin locus were amplified by six multiplex PCR and separated on ABI 3730XL Genetic Analyzer. The signals were then analyzed by the software GeneMapper.

Data Interpretation:

Cell lines were authenticated using Short Tandem Repeat (STR) analysis as described in 2012 in ANSI Standard (ASN-0002) by the ATCC Standards Development Organization (SDO) and in Capes-Davis et al., Match criteria for human cell line authentication: Where do we draw the line? Int J Cancer.2013;132(11):2510-9.

Test Results:

1. Result

Table 2. Matching information on the cell lines

Sample Code	Multi-allele	Cell line matched	Cell Bank	EV	Percentage
20180519-04	Yes	SMMC-7721	ExPasy	1	9/9

- Multi-allele means some STR contain more than two loci.

2. Sample Description

A. The STR results showed that multiple alleles were found in this cell line, and no cross contamination of human cells was found in the cell line.

B. The DNA of the cell lines found to basic match the type of cell lines in a cell line retrieval, DSMZ database shows that cells called **SMMC-7721.** corresponding to the cell number **CVCL_0534, CCRID 3131C0001000700052.**

STR	Amelogenin:X；CSF1PO:10；D13S317:13.3；D16S539:9,10；D18S51:16；D19S433:13；D21S11:27,28；D2S1338:17；D3S1358:15,18；D5S818:1 D7S820:12；D8S1179:12；FGA:18,21；TH01:7；TPOX:12；vWA:16,18

3. Genotyping Result

Table 3. STR and Amelogenin Genotyping Results of Cell line 20180519-04

Loci	**Sample information**			**Cell Bank information**		
	Sample name：7721			Cell line name：SMMC7721		
	**Allele1**	**Allele2**	**Allele3**	**Allele1**	**Allele2**	**Allele3**
D5S818	12	12		12	12	
D13S317	13.3	13.3		13.3	13.3	
D7S820	12	12		12	12	
D16S539	9	10		9	10	
VWA	16	17	18	16	18	
TH01	7	7		7	7	
AMEL	X	X		X	X	
TPOX	12	12		12	12	
CSF1PO	10	10		10	10	
D12S391	20	25				
FGA	18	21		18	21	
D2S1338	17	17		17	17	
D21S11	27	28		27	28	
D18S51	16	16		16	16	

D8S1179	12	12		12	12	
D3S1358	15	18		15	18	
D6S1043	18	18				
PENTAE	7	17				
D19S433	13	13				
PENTAD	8	15				

Others:

1. Genotyping Strategy and Site Distribution

Attached Table. Experimental Strategy and Sites

	Strategy 1	Strategy 2	Strategy 3	Strategy 4
1	TH01	TPOX	D3S1358	AMEL
2	D12S391	VWA	D13S317	D5S818
3	D7S820	D8S1179	D6S1043	D2S1338
4	CSF1PO	PENTAD	D16S539	D21S11
5	FGA		D19S433	D18S51
6	PENTAE			

The allele match algorithm compares the 8 core loci plus amelogenin only, even though alleles from all loci will be reported when available.

2. DSMZ tools was used to carry on the cell line comparison, which contains 2455 cell lines STR data from ATCC, DSMZ, JCRB ,ECACC，GNE and RIKEN databases. If the cell is not included in the above cell library, users need to compared with other databases.

Technician:Menglu Shen
Check: Yang Bai
Person in Charge: Yiqun Chen

Issue date:May 28, 2018
