# Supplementary figures and images for: Synergistic Effect of MC-LR and C-Terminal Truncated HBx on HepG2 Cells and Their Effects on PP2A Mediated Downstream Target of MAPK Signaling Pathway
Source: Front Genet. 2020 Oct 15;11:537785. doi: 10.3389/fgene.2020.537785 (PMC7593820; doi:10.3389/fgene.2020.537785)

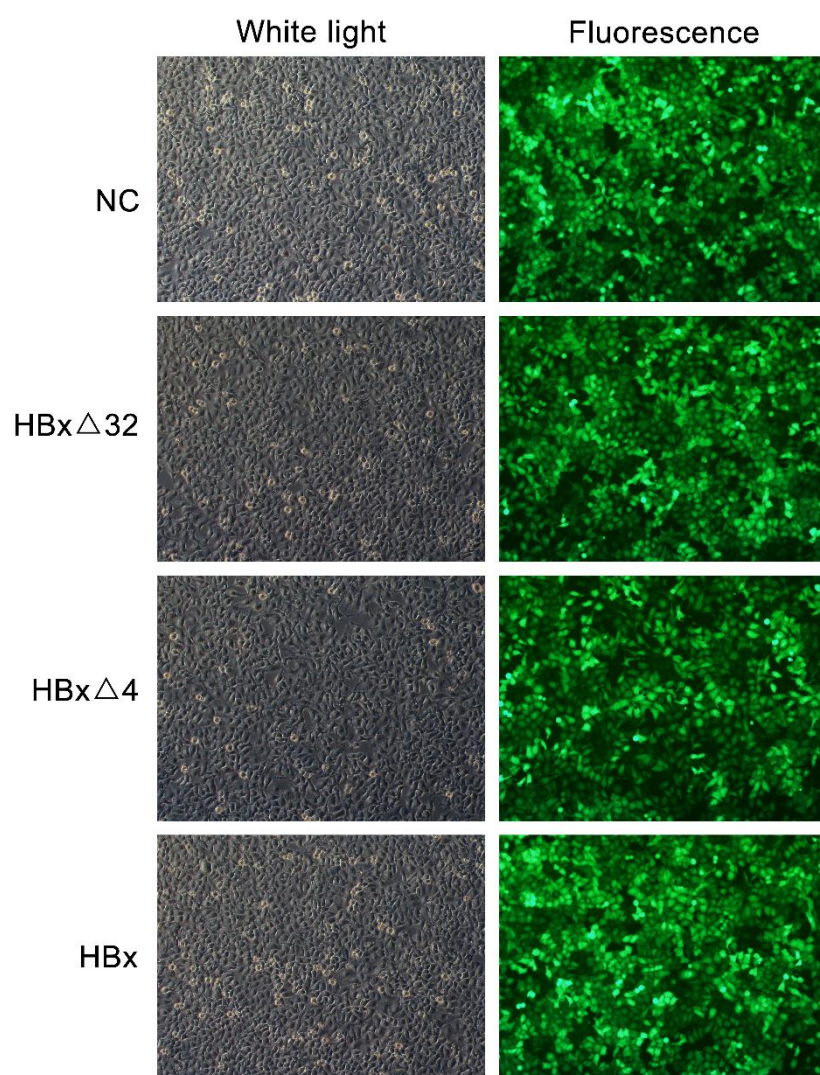

Figure 1 HepG2 cell transfection map ( $\times 100$ )

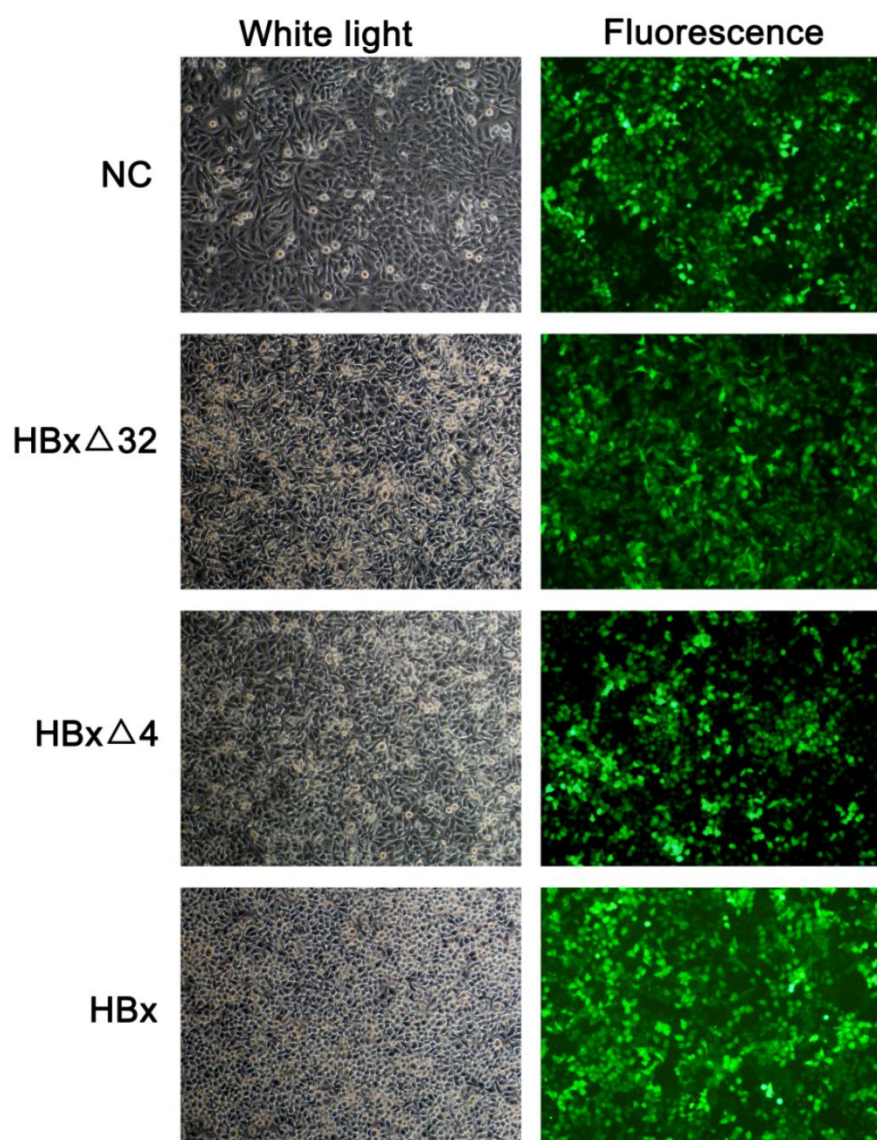

Figure 2 SMMC-7721 cell transfection map ( $\times 100$ )

Supplement: Supplementary file 3 [file Data_Sheet_3.PDF]
