# Supplementary material for: Synergistic Effect of MC-LR and C-Terminal Truncated HBx on HepG2 Cells and Their Effects on PP2A Mediated Downstream Target of MAPK Signaling Pathway
Source: Front Genet. 2020 Oct 15;11:537785. doi: 10.3389/fgene.2020.537785 (PMC7593820; doi:10.3389/fgene.2020.537785)
Supplement: Supplementary file 4 [file Data_Sheet_4.PDF]

Editing Certificate

This document certifies that the manuscript

Synergistic effect of MC-LR and C-terminal truncated HBx on HepG2 cells and their effects on PP2A mediated downstream target of MAPK signalling pathway

prepared by the authors

Chanchan Xiao, Fanbiao Mei, Guanhua Ren, Long Long, Maojian Chen, Xiang Fang, Jilin Li, Kezhi Li, Yanping Tang, Tianren Huang and Wei Deng

was edited for proper English language, grammar, punctuation, spelling, and overall style by one or more of the highly qualified native English speaking editors at AJE.

This certificate was issued on **June 5, 2020** and may be verified on the AJE website using the verification code **5162-D2C4-31B1-C2FF-C2EP**.

Neither the research content nor the authors' intentions were altered in any way during the editing process. Documents receiving this certification should be English-ready for publication; however, the author has the ability to accept or reject our suggestions and changes. To verify the final AJE edited version, please visit our verification page at aje.com/certificate. If you have any questions or concerns about this edited document, please contact AJE at support@aje.com.

AJE provides a range of editing, translation, and manuscript services for researchers and publishers around the world. For more information about our company, services, and partner discounts, please visit aje.com.
