# Supplementary material for: Synergistic Effect of MC-LR and C-Terminal Truncated HBx on HepG2 Cells and Their Effects on PP2A Mediated Downstream Target of MAPK Signaling Pathway
Source: Front Genet. 2020 Oct 15;11:537785. doi: 10.3389/fgene.2020.537785 (PMC7593820; doi:10.3389/fgene.2020.537785)
Supplement: Supplementary file 5 [file Data_Sheet_5.PDF]

Entire gels of the electrophoresis:

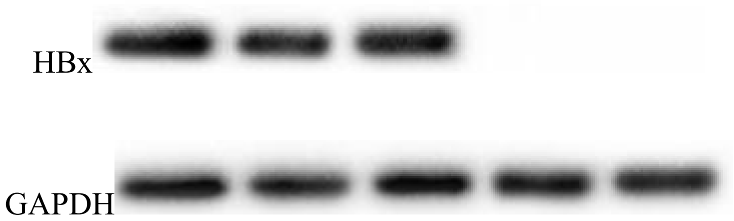

Figure 1 Expression of HBx protein in HepG2 cells

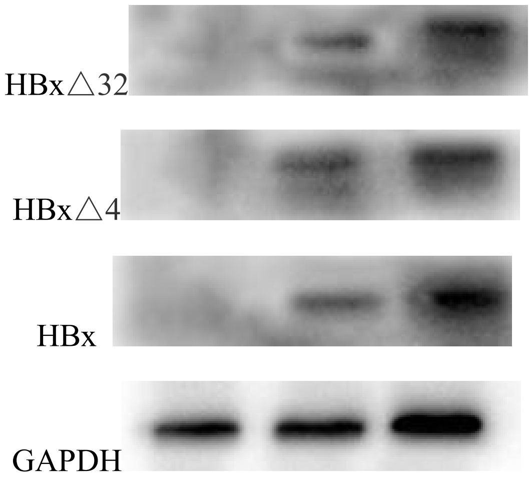

Figure 2 Detection of MC-LR entrance into the HepG2 cells

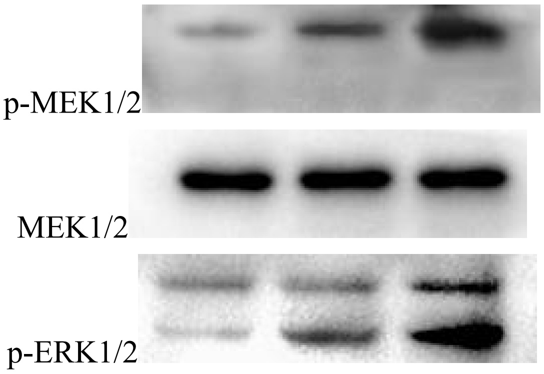

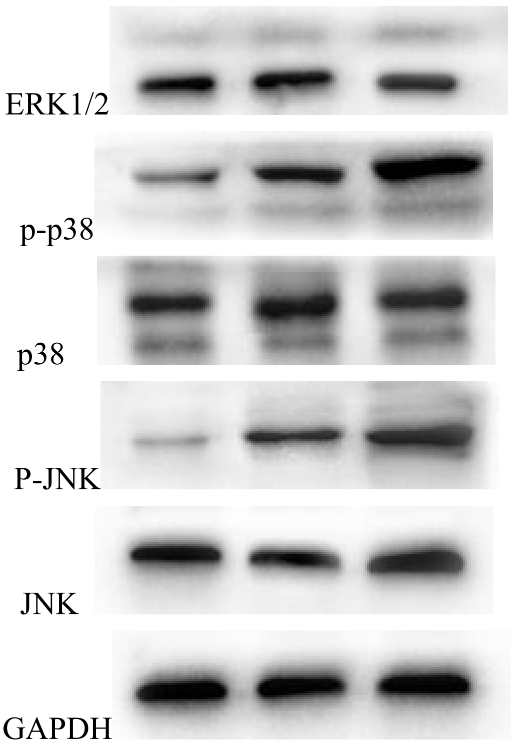

Figure 7 MC-LR and HBxΔ32 activate the MAPK signalling pathway

A

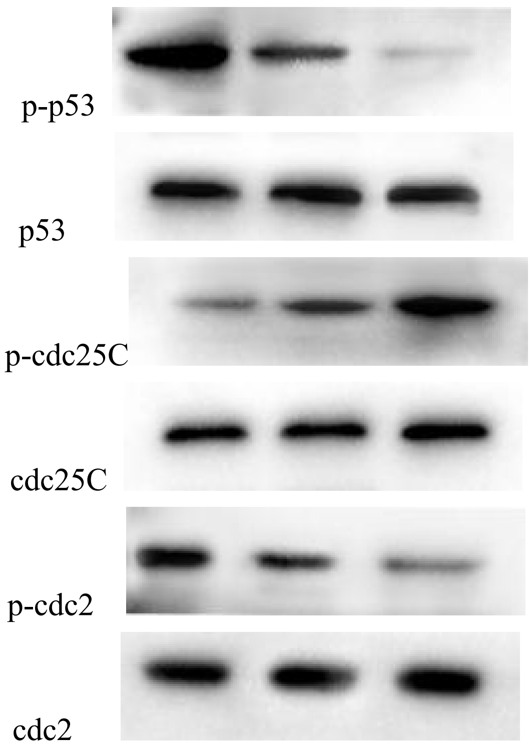

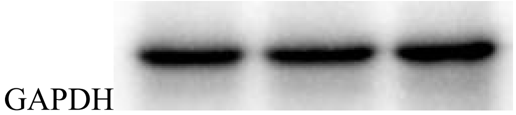

B

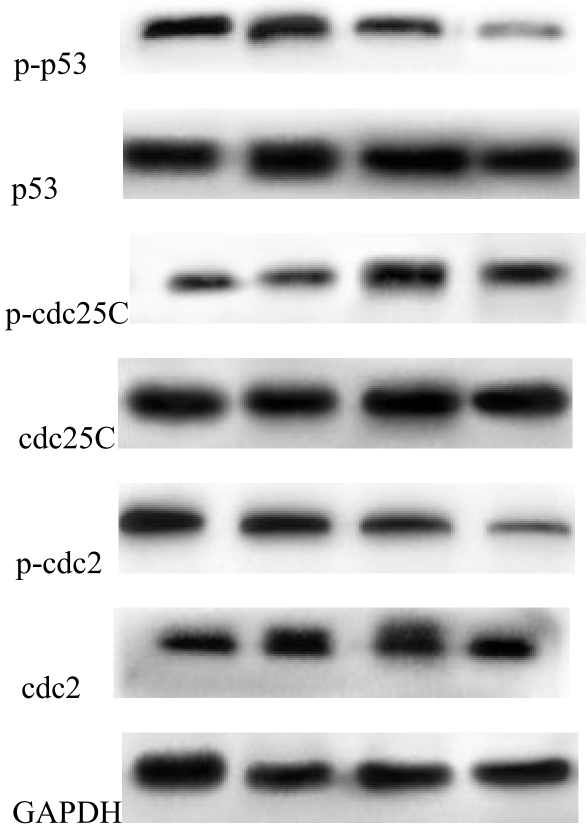

Note:(A)The HBxΔ32 cells were incubated with 10 μM MC-LR for 12 and 24 h.

(B) The cells were pre-incubated with 10 μM DES for 12 h and then exposed to 10 μM MC-LR for 24 h.

Figure 8 MC-LR and HBxΔ32 modulate the phosphorylation of the p53, cdc25C and cdc2 proteins through the activity of PP2A
